# Supplementary material for: Conditional chemoconnectomics (cCCTomics) as a strategy for efficient and conditional targeting of chemical transmission
Source: eLife. 2024 Apr 30;12:RP91927. doi: 10.7554/eLife.91927 (PMC11060718; doi:10.7554/eLife.91927)
Supplement: Figure 3—source data 3. [file elife-91927-fig3-data3.zip › Figure 3 source data3 part 2.zip!/Figure 3-Source data 3-part2/1002-9-CRISPResso_Report_e53dd8m5.zip!/CRISPResso_on_1002_12/9.Alleles_frequency_table_around_sgRNA_gtatctgtccagctacgtga.pdf]

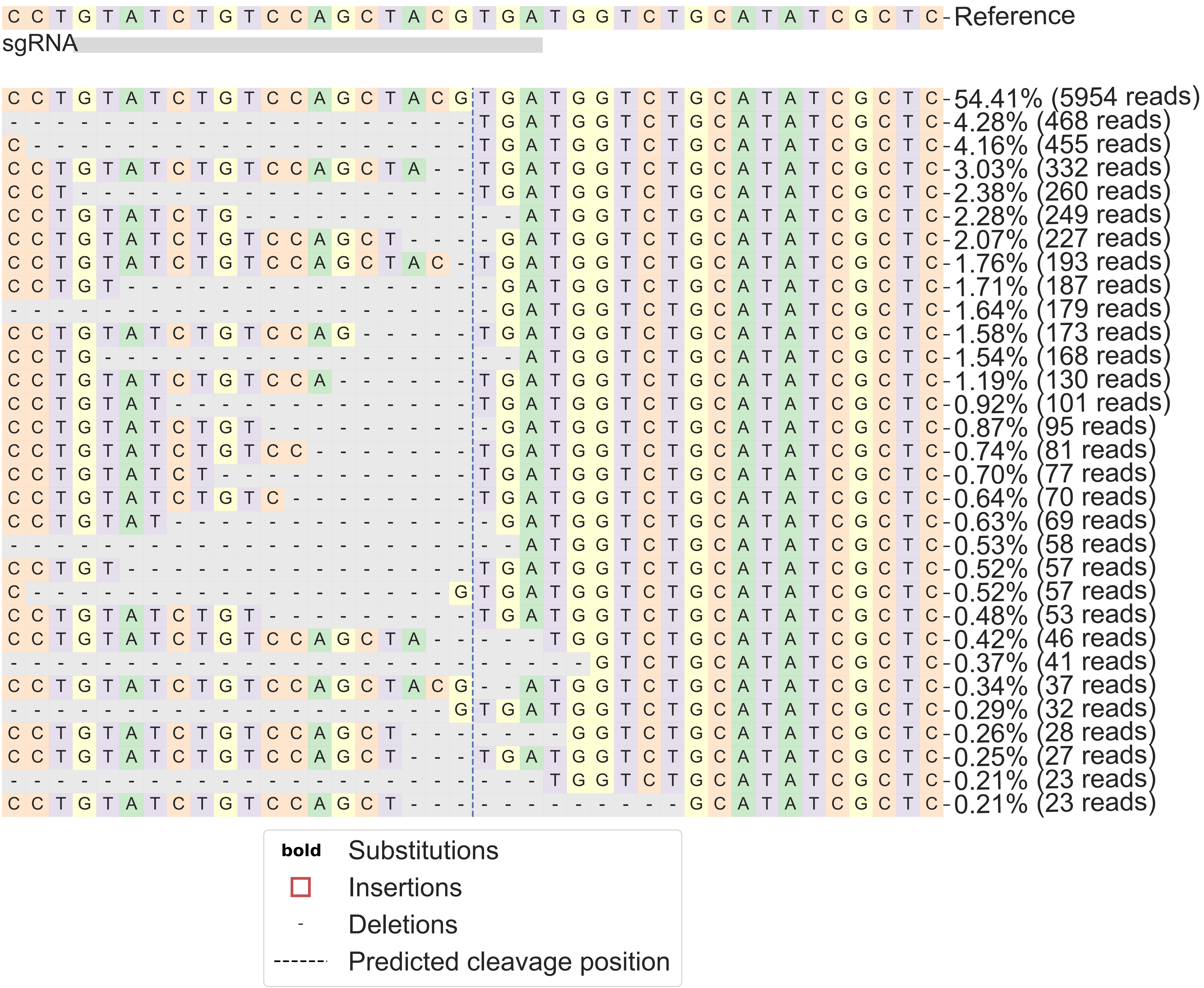
CCTGTATCTGTCCAGCTACGTGATGGTCTGCATATCGCTC Reference
sgRNA
CCTGTATCTGTCCAGCTACGTGATGGTCTGCATATCGCTC 54.41% (5954 reads)
--------------------TGATGGTCTGCATATCGCTC 4.28% (468 reads)
C-------------------TGATGGTCTGCATATCGCTC 4.16% (455 reads)
CCTGTATCTGTCCAGCTA--TGATGGTCTGCATATCGCTC 3.03% (332 reads)
CCT-----------------TGATGGTCTGCATATCGCTC 2.38% (260 reads)
CCTGTATCTG------------ATGGTCTGCATATCGCTC 2.28% (249 reads)
CCTGTATCTGTCCAGCT----GATGGTCTGCATATCGCTC 2.07% (227 reads)
CCTGTATCTGTCCAGCTAC-TGATGGTCTGCATATCGCTC 1.76% (193 reads)
CCTGT----------------GATGGTCTGCATATCGCTC 1.71% (187 reads)
---------------------GATGGTCTGCATATCGCTC 1.64% (179 reads)
CCTGTATCTGTCCAG-----TGATGGTCTGCATATCGCTC 1.58% (173 reads)
CCTG------------------ATGGTCTGCATATCGCTC 1.54% (168 reads)
CCTGTATCTGTCCA------TGATGGTCTGCATATCGCTC 1.19% (130 reads)
CCTGTAT-------------TGATGGTCTGCATATCGCTC 0.92% (101 reads)
CCTGTATCTGT----------GATGGTCTGCATATCGCTC 0.87% (95 reads)
CCTGTATCTGTCC-------TGATGGTCTGCATATCGCTC 0.74% (81 reads)
CCTGTATCT-----------TGATGGTCTGCATATCGCTC 0.70% (77 reads)
CCTGTATCTGTC--------TGATGGTCTGCATATCGCTC 0.64% (70 reads)
CCTGTAT--------------GATGGTCTGCATATCGCTC 0.63% (69 reads)
----------------------ATGGTCTGCATATCGCTC 0.53% (58 reads)
CCTGT---------------TGATGGTCTGCATATCGCTC 0.52% (57 reads)
C------------------GTGATGGTCTGCATATCGCTC 0.52% (57 reads)
CCTGTATCTGT---------TGATGGTCTGCATATCGCTC 0.48% (53 reads)
CCTGTATCTGTCCAGCTA-----TGGTCTGCATATCGCTC 0.42% (46 reads)
-------------------------GTCTGCATATCGCTC 0.37% (41 reads)
CCTGTATCTGTCCAGCTACG--ATGGTCTGCATATCGCTC 0.34% (37 reads)
-------------------GTGATGGTCTGCATATCGCTC 0.29% (32 reads)
CCTGTATCTGTCCAGCT-------GGTCTGCATATCGCTC 0.26% (28 reads)
CCTGTATCTGTCCAGCT---TGATGGTCTGCATATCGCTC 0.25% (27 reads)
-----------------------TGGTCTGCATATCGCTC 0.21% (23 reads)
CCTGTATCTGTCCAGCT------------GCATATCGCTC 0.21% (23 reads)
bold Substitutions
Insertions
Deletions
Predicted cleavage position
